# Use of Meta-analyses by IARC Working Groups

**DOI:** 10.1289/ehp.1205397

**Published:** 2012-08-31

**Authors:** Kurt Straif, Leslie Stayner, Paul A. Demers, Philip J. Landrigan

**Affiliations:** 1 International Agency for Research on Cancer, Lyon, France, E-mail: straif@iarc.fr; 2 School of Public Health, Division of Epidemiology and Biostatistics, University of Illinois at Chicago, Chicago, Illinois; 3 Occupational Cancer Research Centre, Toronto, Ontario, Canada; 4 Department of Community and Preventive Medicine, Mount Sinai School of Medicine, New York, New York

In their letter, Kogevinas and Pearce (2012) suggested that meta-analyses should be more routinely prepared for the evaluations of the International Agency for Research on Cancer (IARC) Monographs program. We concur that meta-analyses are useful in many cases, but there are also counter examples where they have not been useful. For example, when Kogevinas et al. (1998) reviewed the carcinogenicity of cancer hazards in the rubber-manufacturing industry, they argued against using meta-analytic techniques because of the heterogeneity of exposure circumstances within and between manufacturing plants and differences of exposure classifications used in the studies. They concluded that a single summary risk estimate would be uninformative. Based on their systematic narrative review, the authors concluded that there is an increased risk of neoplasms of the urinary bladder, lung, and larynx and an increased risk of leukemia (Kogevinas et al. (1998). In contrast, Alder et al. (2006) performed a meta-analysis of cancer occurrence among workers in the rubber-manufacturing industry. Based on summary estimates for the entire rubber industry and two major sectors of this industry, these authors concluded that excesses other than for leukemia were not substantiated by their synthetic meta-analysis (Alder et al. 2006). After reviewing all the pertinent studies, a later IARC Working Group concluded that there is sufficient evidence for an increased risk of several types of cancer in rubber manufacturing (Baan et al. 2009).

In contrast, when the IARC Working Group for Volume 98 reviewed the evidence on shift work and cancer, a published meta-analysis had reported a statistically significantly increased risk for breast cancer among women who regularly worked the night shift (Megdal et al. 2005). Nevertheless, the IARC Working Group concluded that there was only limited evidence for carcinogenicity in humans (IARC 2010).

In the context of the Volume 98 Monographs meeting, the Working Group performed a meta-analysis and concluded that there was sufficient evidence for the carcino-genicity of exposures as a painter (IARC 2010). In preparation for the Volume 100 series of the *IARC Monographs*, this meta-analysis was further developed, taking into account studies published after the Volume 98 meeting (Guha et al. 2010). This meta-analysis and another one (Bachand et al. 2010) were available to the Working Group for Volume 100F. Bachand et al. (2010) did not provide results by duration of employment or for nonsmokers, but they argued that the increased risks could be due to residual confounding. After reviewing all published evidence, the IARC Working Group reconfirmed the carcinogenicity of exposures as a painter.

In general, during the last two decades meta-analyses have become more widely used in epidemiology, and the 2006 amendment of the IARC Preamble now specifically mentions the possibility of premeeting and ad hoc meta-analyses during the course of a Monograph meeting (IARC 2006). In practice, this has been done even earlier, for example, when the Working Group for Volume 83 updated a published meta-analysis on involuntary smoking and lung cancer (IARC 2004). Anticipating scenarios as described above, the Preamble (IARC 2006) stresses the need “that the same criteria for data quality be applied as those that would be applied to individual studies.”

Kogevinas and Pearce (2012) referred to a recently published meta-analysis for asbestos and ovarian cancer that we coauthored (Camargo et al. 2011). Interestingly, another meta-analysis of this same question was published by Reid et al. (2011). Whereas our meta-analysis focused on occupational cohorts with well-documented exposure to asbestos and identified almost twice as many cases from occupational cohorts, Reid et al. also included environmental and household exposures as well as linkage and case–control studies. Nevertheless, both meta-analyses reported increased risks overall and in most stratified analyses. However, while Reid et al. (2011) believed that increased risks may be due to disease misclassification, we (Camargo et al. 2011) concluded that our meta-analysis supports the IARC classification. This illustrates again that meta-analyses are not free from subjective decisions and interpretations.

In conclusion, meta-analyses are a quantitative statistical tool that, in some instances may inform causal inference, but they never alleviate the need for critical review of all available data; narrative reviews by an interdisciplinary IARC Working Group may be, in some cases, more informative than a synthetic meta-analysis. Therefore, although a comprehensive review of all original data is required, a comprehensive review of all meta-analysis may not be warranted, particularly when the meta-analyses are outdated or cover only a subset of the original studies. The current “Preamble to the *IARC Monographs”* (IARC 2006) provides the Working Group with all options to perform quantitative meta-analysis where appropriate and helpful for causal inference. Different approaches have been applied in the history of the *IARC Monographs*. The Volume 100 series of the *IARC Monographs* confirmed all Group 1 carcinogens identified during the 40-year history of the monographs, which in turn confirmed that the procedures of the *IARC Monographs* are robust. With more epidemiological studies becoming available for each agent, additional cancer sites being investigated, and relatively small effect estimates becoming center of the discussion, the need for meta-analyses is likely to increase.
